# Deep Learning Methods for Space Situational Awareness in Mega-Constellations Satellite-Based Internet of Things Networks

**DOI:** 10.3390/s23010124

**Published:** 2022-12-23

**Authors:** Federica Massimi, Pasquale Ferrara, Francesco Benedetto

**Affiliations:** 1 Signal Processing for Telecommunications and Economics Laboratory, Roma Tre University, 00145 Rome, Italy; 2 Leonardo Labs, Leonardo S.p.a., 00131 Rome, Italy

**Keywords:** deep learning, object detection, Satellite IoT, space surveillance awareness

## Abstract

Artificial Intelligence of things (AIoT) is the combination of Artificial Intelligence (AI) technologies and the Internet of Things (IoT) infrastructure. AI deals with the devices’ learning process to acquire knowledge from data and experience, while IoT concerns devices interacting with each other using the Internet. AIoT has been proven to be a very effective paradigm for several existing applications as well as for new areas, especially in the field of satellite communication systems with mega-constellations. When AIoT meets space communications efficiently, we have interesting uses of AI for Satellite IoT (SIoT). In fact, the number of space debris is continuously increasing as well as the risk of space collisions, and this poses a significant threat to the sustainability and safety of space operations that must be carefully and efficiently addressed to avoid critical damage to the SIoT networks. This paper aims to provide a systematic survey of the state of the art, challenges, and perspectives on the use of deep learning methods for space situational awareness (SSA) object detection and classification. The contributions of this paper can be summarized as follows: (i) we outline using AI algorithms, and in particular, deep learning (DL) methods, the possibility of identifying the nature/type of spatial objects by processing signals from radars; (ii) we present a comprehensive taxonomy of DL-based methods applied to SSA object detection and classification, as well as their characteristics, and implementation issues.

## 1. Introduction

Artificial Intelligence (AI) is now a part of the daily life of the majority of people. AI is based on the idea that computers can (be programmed to) think just like humans, creating analyses, reasoning, understanding, and getting answers for and from different situations. The big step within the studies of AI is the development of systems capable of learning and developing on their own, or of creating new deductions from the junction of various fragmented information, just as happens within the neurological system of human beings. Therefore, AI is the technology that provides intelligent machines to solve problems, increase productivity, and improve areas such as health, finance, marketing, sales, customer service, and agriculture, among many other fields of application [1,2,3].

The Internet of Things (IoT), on the other hand, is a world with smart technology that impacts almost every aspect of our society. When we think about IoT, we think about technological tools used in everyday life, such as connected thermostats, home security systems, and cars [4,5,6]. The Artificial Intelligence of Things (AIoT) is the sweet spot that combines the best of both worlds: it leverages the technical capabilities of the IoT and makes the best use of the data processing and interpretation capabilities of AI to deliver advanced solutions to users, meeting their specific needs and use cases [7,8]. One of the main benefits of AI in action in an IoT environment is real-time decision-making. The results are obtained instantaneously. For example, an AI-based surveillance system with object detection technology can instantly detect work or environmental hazards, such as sparks from equipment or an employee’s fall, and immediately report notifications. Advanced systems can even make decisions and trigger incident responses on their own [9,10,11].

The IoT technology is as promising as it can be, but it is not without its fair share of implementation shortcomings and challenges. Simplifying data from multiple sources is challenging; one needs to work on infrastructure requirements to examine data security and privacy, etc. The recent scientific and technological advances led by AIoT have also allowed for many opportunities in the space industry, which is a type of field that has been increasingly developed over the last decade, and very significant benefits are expected soon in the field of Satellite for IoT (SIoT). In the harsh industrial environment, the necessity for joining IoT and satellite communications is needed [12]. Many studies have been carried out, especially for low-orbit satellites, due to their interesting characteristics, such as low latency and large capacity [13,14,15,16,17]. As a consequence, IoT applications through satellite systems are developing quickly and promise to be the biggest prospect for the satellite market of the future.

**Figure 1:**
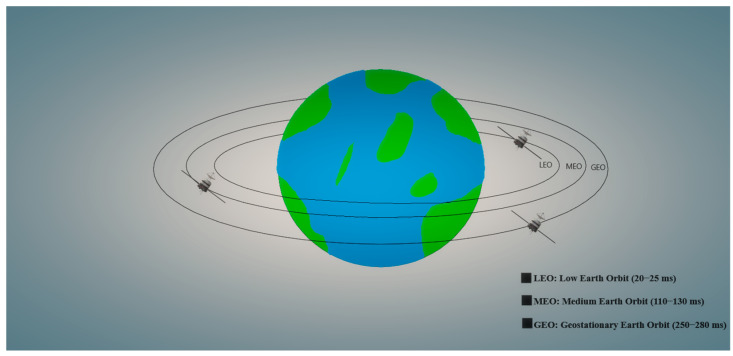
Different types of satellite orbits around the Earth.

IoT applications will be very well supported by satellite mega-constellations. These are systems with thousands, and even tens of thousands, of satellites in low Earth orbit (LEO), and they are nowadays becoming a reality [18,19,20]. Among all of Earth’s orbital regimes, LEO, generally defined as the region in space between ~160 to 2000 km in altitude [21], is by far the most congested (Figure 1). As the closest to Earth and, therefore, the cheapest to reach, LEO is the most popular orbital regime for satellite deployments [22]. Companies are placing satellites into orbit at an unprecedented frequency to build mega-constellations of communications satellites in LEO. In two years, the number of active and defunct satellites in LEO has increased by over 50% to about 5000. SpaceX alone is on track to add 11,000 more as it builds its Starlink mega-constellation and has already filed for permission with the Federal Communications Commission (FCC) for another 30,000 satellites [23]. Others have similar plans, including OneWeb, Amazon, Telesat, and GW, a Chinese state-owned company. Thousands of satellites and 1500 rocket bodies provide considerable mass in LEO, which can break into debris upon collisions, explosions, or degradation in the harsh space environment. Fragmentations increase the cross-section of orbiting material and with it, the collision probability per time. Eventually, collisions could dominate on-orbit evolution, a situation called the Kessler Syndrome [24]. 

There is much debris or “space junk” in the near-Earth space environment that is large enough to threaten human spaceflights and robotic missions but too small to be tracked. The International Space Station (ISS) and other spacecraft with humans on board, due to the exponential growth of space debris, could be endangered. This debris population includes both natural meteoroids and man-made orbital debris. This debris population includes both natural meteoroids orbiting the sun, and man-made orbital debris, orbiting the Earth, called “orbitals”. Orbital debris generally is any man-made object in Earth’s orbit that no longer serves a useful function, such as non-functioning spacecraft, abandoned launch vehicle stages, mission-related debris, and fragmentation debris. They are constantly tracked, and more than 27,000 pieces have been detected so far. Since both debris and spacecraft travel at extremely high speeds (about 25,267 km/h), the impact of collision of even a tiny piece of orbital debris with a spacecraft could create serious and dramatic problems. There are half a million pieces of debris the size of a marble or larger (up to 0.01016 m, or 1 cm), and about 100 million pieces of debris about 0.001016 m (or one millimeter) and larger. There is also even smaller micrometer-sized debris (9.906 × 10^−7^ m in diameter) and about 23,000 pieces of debris larger than a softball orbiting the Earth. Even small flecks of paint can damage a spacecraft when traveling at these speeds. For example, many space shuttle windows have been replaced due to some damage caused by paint stains. Simulations of the long-term evolution of debris suggest that LEO is already in the protracted initial stages of the Kessler Syndrome but that this could be managed through active debris removal [25]. The addition of satellite mega-constellations and the general proliferation of low-cost satellites in LEO stresses the environment further [26,27,28]. Hence, decades of the world’s space activities have left LEO cluttered with active satellites and littered with orbital debris. Recent reports [29,30] estimate that there are about 28,210 debris objects regularly tracked by Space Surveillance Networks. While large commercial satellite constellations such as SpaceX’s Starlink undeniably offer tremendous potential for the satellite industry, they inevitably increase the probability of mutual collisions among orbiting objects due to the inherently high number of satellites involved in large constellations. This poses a significant threat to the sustainability and safety of space operations that must be carefully and efficiently addressed.

**Figure 2:**
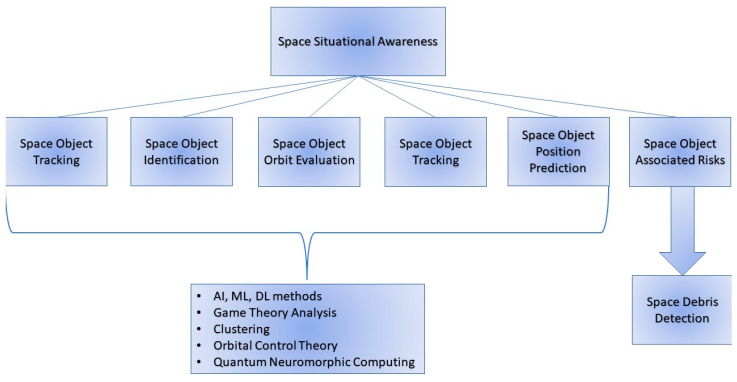
Space Situational Awareness: objectives and enabling technologies.

The operation of monitoring the space environment and resident space objects, knowing and characterizing space objects and their operational environment, is known as space situational awareness (SSA) [31]. SSA is a fundamental part of Space Domain Awareness (SDA), which represents the capacity of understanding the actual and expected working conditions in space. In more detail, SSA focuses on the problems related to (i) space object tracking, (ii) identification, (iii) determining their orbits, (iv) gaining knowledge about the scenario in which they are working, and (v) forecasting their upcoming positions and risks to their functioning (Figure 2). SSA is hence of fundamental importance to all space traffic management (STM) operations, and one of the fundamental aspects of SSA is to calculate and act in response to debris from fragmentation events, meteor storms, or other natural events that will be very dangerous for all the space systems. One critical task is to classify space objects according to their properties. Unfortunately, the information on space objects is often limited. Typically, the visual magnitude and the radar cross section (RCS) of space objects can be obtained via optical and radar sensors, respectively. Artificial Intelligence (AΙ) and machine learning (ML) systems appear to be very promising for detecting and classifying these kinds of objects. Methods for assessing behavioral analysis [32] and autonomy [33,34], reported in the literature, include Artificial Neural Networks (ANN) [35,36], Support Vector Machines (SVM) [37], reinforcement learning [38,39], and deep neural networks [31]. 

These ML methods, as well as deep learning (DL) methods [40], support evidence-based knowledge for SDA [41] of space-domain sensor fusion programs such as DARPA Hallmark [42]. SSA also includes the understanding of mission policies, technical aims, and orbital mechanics [43,44,45]. In addition, SSA benefits from game-theory studies in supporting pursuit-evasion analysis [46,47,48,49,50] and from gathering data to track satellites, debris, and natural phenomena [51,52,53,54,55,56,57]. Then, in order to have effective SSA results, tracking efforts must coordinate with detection policies [58,59], waveform selection [60], and attack mitigation [61,62,63,64]. 

Other recent methods for effective SSA include time-delay Neural networks (TDNN) [65], ML and CNN networks [66,67,68,69,70], clustering [71], orbital control theory [72], a game theoretic approach, namely Adaptive Markov Inference Game Optimization (AMIGO) engine [73,74,75,76], and Deep Reinforcement Learning [77,78,79]. Even more recently, new trends in the literature are focusing on agile, intelligent, and efficient computer vision architectures operating on quantum neuromorphic computing as part of an SSA network [80]. Quantum neuromorphic vision, in conjunction with polarimetric Dynamic Vision Sensors p(DVS) principles, represent the SSA of tomorrow, working at very high speeds with low requirements for bandwidth, power, and memory.

In this paper, we will focus on and review methods belonging to a specific subgroup of SSA, namely on space debris automatic detection, since millimeter-sized orbital debris poses the highest end-of-mission risk for most spacecraft roboticists operating in LEO [81,82]. In this context, this work aims at providing an overview of recent AI and AIoT for space applications by examining the current state of the art and discussing future opportunities to support efforts in space exploration. The rest of our paper is organized as follows. In Abbreviations, a list of the used acronyms is reported. Section 2 is divided into three sub-sections and illustrates SSA space debris applications, with and without the use of deep learning methods. The last part of Section 2 shows some interesting discussions about the use of new types of neural networks. Subsequently, in Section 3, we will discuss the case study and the experimental setup. The results, used as a proof of concept of some of the reviewed DL methods for space debris detection, are then shown and discussed in Section 4, while Section 5 briefly concludes our work, outlining the strong and weak points of these technologies.

## 2. Related Works

**Figure 3:**
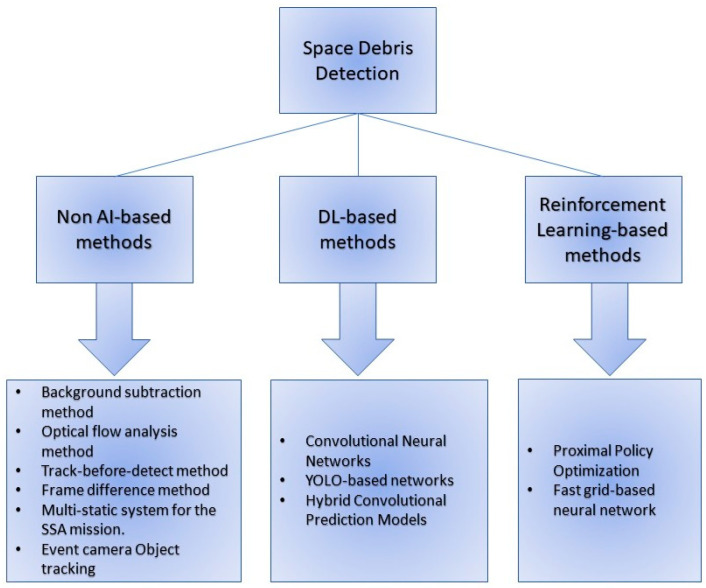
Space debris detection as a sub-group of SSA: enabling technologies.

According to the literature, and to the best of the authors’ knowledge, the published works on space debris detection can be categorized into three groups (Figure 3): (i) non-AI-based methods, (ii) DL-based methods, and (iii) Reinforcement learning-based methods. Hence, in the following, the survey is divided into three parts accordingly.

### 2.1. Non-AI-Based Methods

In the past years, algorithms for the detection of small space objects have been extensively studied and can be categorized as follows:

- Background subtraction methods [83,84,85] are one of the most popular approaches for detecting objects. This algorithm works by comparing the moving parts of a video with a background and a foreground image.
- Optical flow analysis method [86] is a technique used to describe the movement of objects within a sequence of images that have a small-time gap between them, such as video frames. Optical flow calculates a motion vector for the points within the images and provides an estimate of where the points might be in the next image.
- Track-before-detect methods [87,88] is a concept where a target is tracked before it is detected. Usually, target detection is performed by means of thresholding of the input signal, and the output is passed to a tracker. In this paradigm, sensor data on a provisional target are integrated over time, and the target is detected without the use of any threshold. This approach is able to track targets even with low SNRs (signal-to-noise ratios).
- Frame difference methods [89,90] are similar to the background subtraction methods. This is a technique where the system checks, pixel-by-pixel, the difference between two consecutive video frames. If two corresponding pixels have changed their appearance, motion is detected.

Most of these techniques work well with a little blur and thresholds to distinguish real motion from noise because frames could significantly differ from each other as, for example, lightning and conditions change. Other types of object-tracking methods have recently been developed. In [91], the goal was to evaluate the performance of a multi-static radar for the SSA mission, also presenting the advantages and disadvantages of the use of telescopes or radars. The advantage of using optical telescopes allows you to observe objects at very long distances compared to radar observations. On the other hand, radar systems can operate 24/7 regardless of weather conditions and do not depend on sunlight as a source of illumination. They can fine-tune the transmitted signal to perform proper processing and estimate the physical and dynamic properties of the target, providing highly accurate measurements essential for orbit determination techniques. However, one of the major disadvantages of using radar for SSA purposes is its high cost, as such systems require very high transmitting power. 

We can see another type of object tracking experiment in [92] where a camera is used for events. When a pixel emits an event, the current brightness is stored and continuously monitored for changes in brightness. If the modified value exceeds a threshold, the camera generates an event. When the changed value exceeds a threshold, the camera generates an event containing the pixel coordinates (x, y), time (t), and the changed polarity (p) (+1 for brightness increasing, −1 for brightness decreasing). There are many benefits to using this method, including the following:

- High temporal resolution: unlike conventional cameras, event cameras can catch extremely fast motion without experiencing motion blur
- Low Latency: there is no need to wait for the frame’s overall exposure time because each pixel operates independently. An event is notified as soon as the change is discovered.
- Low Bandwidth: by broadcasting brightness changes, event cameras eliminate duplicate data.
- Low Power: in event cameras, power is only utilized to process the shifting pixels.
- Despite the advantages listed above, there are also significant disadvantages:
- High Noise: because of how the sensors are built, event cameras are particularly susceptible to background activity noise brought on by transient noise and leakage currents from semiconductor PN junctions.
- Large Pixel Size: compared to a regular camera, the modern event camera has larger pixels. The event camera’s resolution is relatively low due to the high pixel size.
- Low Fill Factor: the fill factor of event cameras is usually small, which means that a lot of pixel area is useless.

In Table 1, we summarize the methods, their characteristics, and the bibliographic references. 

**Table 1:** Non-AI-based methods for SSA space debris detection.

Method Name	Description	Reference Number
Background subtraction method	This paper introduces background subtraction methods and reports a comparison of the most promising cutting-edge algorithms.	[83]
	This work provides a specific perspective view on background subtraction for detecting moving objects, as a building block of many computer vision applications, being the first relevant step for subsequent activity recognition, classification and analysis.	[84]
	This article introduces a background subtraction algorithm for detecting fast moving objects. In particular, the algorithm proved effective in detecting change in global illumination, static foreground objects, camouflaged objects, ghosts and dynamic background compared to seven other cutting-edge methods.	[85]
Optical flow analysis method	This article presents an experiment to verify the accuracy of the optical flow method (i.e., the apparent movement of individual pixels in the image plane). The accuracy of the technique is evaluated for different amplitudes of sub-pixel vibration displacement.	[86]
Track-before-detect method	This paper addresses the detection and monitoring of weak and maneuvering targets using the MF-TBD (Multi-Frame Track-Before-Detect) method.	[87]
	This work considers the underwater tracking of an unknown and time-varying number of targets, for example acoustic emitters, using passive array sonar systems	[88]
Frame difference method	This article introduces the problem of moving target detection in video sequences. An improved frame difference target detection algorithm is proposed on the basis of the self-updating medium background model.	[89]
	A motion detection application with the frame difference method on a surveillance cameras CCTV (Close-Circuit Television) is discussed.	[90]
Multi-static system for the SSA mission.	The main objective of this article is to evaluate the performance of a multi-static system for the SSA mission. In addition, a simulation tool was developed to test the performance in different scenarios.	[91]
Event camera Object tracking	This article first introduces the basic principles of the event camera, then analyzes its advantages and disadvantages.	[92]

### 2.2. DL-Based Methods

AI automation can be very useful for optimizing the large amount of data collected by scientific missions such as deep space probes, Earth observation spacecraft, and rovers. It also helps in evaluating data and disseminating the results to the end users. By employing AI aboard spacecraft, it will be possible to build datasets and maps, all independently. AI technology is not only capable of processing a large amount of information but can minimize or eliminate redundant data (e.g., compression) so that networks can operate more efficiently. Artificial Intelligence also enables a variety of monitoring tasks thanks to ubiquitous satellite imagery in space. Deep learning (DL), as a subfield of Artificial Intelligence, can enable precise and automated control and facilitate onboard activities, such as docking or navigation [93,94,95]. 

The rationale behind this meticulous DL hype is the weakness of traditional ML techniques in meeting the growing analytical requirements of IoT systems. DL models (especially CNN) are superior to traditional ML techniques in several ways: first, compared to ML techniques, they require little or no pre-processing steps to alleviate the prerequisite that tagged data must be used for training. Thus, features that may not be identifiable to a human can be efficiently extracted from DL approaches. Furthermore, these approaches outperform traditional techniques in terms of accuracy. Finally, DL architectures are suitable for modeling complex behaviors of conventional multimodal datasets. So, in summary, DL models can work on any data, be it structured, unstructured or semi-structured [96]. 

The rest of this section will focus on several DL-based methods, namely CNN and YOLO (You Look Only Once) networks, and on hybrid convolutional prediction models. Accordingly, in the following, we have three sub-sections. Finally, in Table 2, we summarize the methods, their characteristics, and the bibliographic reference.

#### 2.2.1. CNN-Based Methods

Convolutional neural networks have proven their worth in the areas of image recognition and classification. They are classified as deep learning (DL) algorithms, which take digital signals such as audio, images, and videos as inputs. They reached outstanding results in the field of image classification. Images go through discrete convolutional layers aiming to extract different aspects or features of the image and to assign them weights and biases to classify them. Different types of solutions have been implemented for tracking and classifying objects. In [97], the authors propose a method for detecting the salience of space debris based on a fully convolutional network (FCN) for the space surveillance platform. At the same time, the network directly learns the internal relationship between two frames, thus avoiding expensive optical flow calculations. However, the method is unsuitable for detecting small targets due to the limited features extracted from small space debris. In [98], a novel U-Net deep neural network approach is exploited for image segmentation for real-time extraction of tracklets from optical acquisitions. Artificially tagged images and synthetic footage of the night sky of objects passing over a specified location were artificially generated, and the corresponding labels were produced—stars appear as points in the field of view (FoV), while debris appears as streaks (usually called “tracklet”), resulting in a bright streak of pixels on the image. After a pre-processing step, the actual images were optimized for vignetting reduction and background brightness uniformity. In object recognition and classification, convolutional neural networks outperform not only any of the techniques mentioned above but also any ML technique, such as demonstrated in the work [99]. Here, the authors obtained the best accuracy of the detected image objects by using intelligent surveillance cameras. The use of hierarchical MCNNT (Modified Convolutional Neural Networks Techniques) models of data processing modification improves the performance of CNN classification, reaching 96% of accuracy against existing support vector machine (SVM) models. 

A new type of convolutional network is presented in [100]. PSnet is a perspective-sensitive network to detect objects from different perspectives (i.e., angles of view). The features are mapped to the preset multi-perspective spaces to obtain the specific semantic feature of the object decoupled from the angle of view. With the help of PSnet, the overall separability of functionality is improved as objects between classes are projected onto different segments, and objects between classes with different viewing angles are projected onto the same segments. The work [101] proposes an end-to-end spacecraft image segmentation network using the DeepLabv3+ semantic segmentation network as the basic framework. A multiscale neural network based on sparse convolutions (SISnet) is developed. The innovation of this method is twofold—firstly, the ability to extract features is better from the dilated convolutional network, and secondly, the channel attention mechanism is introduced into the network to recalibrate the functionality responses. Finally, a parallel spatial pyramid pooling (ASPP) structure is designed to improve the contextual information of the network. To verify the effectiveness of the method, experiments are conducted on a spacecraft segmentation dataset. The experimental results show that the encoder + attention + decoder structure proposed in this paper can obtain clear and complete masks of spacecraft targets with high segmentation accuracy. In [31], The authors explore DNN (deep neural networks) using light curve data. Conventional classification algorithms, such as k-nearest neighbor (k-NN), are implemented and compared in terms of accuracy with the proposed DNN-based classification algorithms, including the popular CNN and the recurrent neural network (RNN). The concept of an RNN is different from CNN. The hidden level of RNN is time-related. For time series data, the input is processed sequentially from different levels. Originally, RNN had the vanishing gradient problem, which meant that the gradient became extremely small with the number of propagation layers. This implies that network weights might not be updated during the learning process. To solve the problem, long-short-term memory (LSTM [102]) has been proposed, which can be seen as an improvement to solve the original RNN problem. Both CNN and RNN show promising results over the conventional k-NN-DTW (k-nearest neighbor combined with Dynamic Time Warping) algorithm [31]. Two-dimensional CNN also shows promising results, providing an alternative way to use time series data directly. 

#### 2.2.2. YOLO Networks

The YOLO algorithm uses neural networks to deliver real-time object detection, performing object detection as a regression problem. Because of its precision and velocity, Yolo is used in many object detection applications. In [103], a simple framework for recognizing moving objects is proposed. The framework consists of two steps that are applied sequentially to each video frame. In the first step, using simple background subtraction, regions of interest (ROIs) are found in a video frame. In the second step, a YOLO network (namely YOLOv2 [104]) is used to rank the detected ROIs based on a set of predefined criteria. The framework offers the advantage of reusing background subtraction. In fact, the real processing time dedicated only to the detection of objects in the framework is the CNN inference time. YOLO is also used for machine vision target detection of unmanned aerial vehicles (UAVs). YOLOv3-Tiny optimizes the YOLOv3-based network structure and reduces the output by one scale. In this work, human images are used to train YOLOv3-Tiny to recognize humans as tracking targets. Then, YOLOv3-Tiny analyzes the images collected by the UAV for target tracking. Finally, the tracking system control signal is sent to the flight controller of the UAV [105].

#### 2.2.3. Hybrid Convolutional Prediction Models

In [106], a new model for zero-click action recognition is studied, which jointly captures the object relations of a static frame and models the temporal motion patterns of adjacent frames. The object detector first detects and extracts the characteristics of the object. The convolutions of the graph are then conducted to effectively exploit object relationships. The work presented in [107] provides a way of planning the use of solar energy. Accurate production forecasts are essential for photovoltaic projects to achieve stable power. Traditional forecasts based on soil observation time series are largely troubled by the problem of phase shift due to incomplete consideration of the impacts of cloud movement. This work develops an innovative framework that integrates ground-based and satellite observations through DL to improve photovoltaic production forecasts. The framework is based on a hybrid deep-prediction model to account for the impact of cloud movements on subsequent changes in solar radiation, a geometric calculator to simulate the relative position between the Sun and photovoltaic panels, and a photovoltaic estimator to model the performance of photovoltaic modules during photoelectric conversions.

**Table 2:** DL-based methods for SSA space debris detection.

Method Name	Description	Reference Number
Convolutional Neural Networks	This paper shows that DL architectures are suitable for modeling complex behaviors of conventional multimodal datasets. It is demonstrated that DL models have the ability to work on any data, be it structured, unstructured or semi-structured	[96]
	The authors propose a method for detecting the salience of space debris based on a fully convolutional network (FCN) for the space surveillance platform.	[97]
	A novel U-Net deep neural network approach is exploited for image segmentation for real-time extraction of tracklets from optical acquisitions	[98]
	The use of hierarchical MCNNT (Modified Convolutional Neural Networks Techniques) improves the performance of CNN classification, reaching a 96% accuracy against existing support vector machine (SVM) models.	[99]
	PSnet is a perspective sensitive network to detect objects from different perspectives (i.e., angles of view). The features are mapped to the preset multi-perspective spaces to obtain the specific semantic feature of the object decoupled from the angle of view.	[100]
	An end-to-end spacecraft image segmentation network using the DeepLabv3 + semantic segmentation network as the basic framework is proposed here. Then, a multiscale neural network based on sparse convolutions (called SISnet) is developed.	[101]
	Conventional classification algorithms, such as k-nearest neighbor (k-NN), are implemented and compared in terms of accuracy with the proposed DNN-based classification algorithms, including the popular CNN and the recurrent neural network.	[31]
YOLOv3-based networks	A simple two-step framework for recognizing moving objects is proposed. In the first step, regions of interest (ROIs) are found in a video frame. In the second step, a CNN is used to rank the detected ROIs based on a set of predefined criteria.	[103]
	YOLOv3-Tiny optimizes the YOLOv3-based network structure and reduces the output by one scale. Human images are used to train YOLOv3-Tiny so that they can recognize humans as tracking targets. Then, YOLOv3-Tiny analyzes the images collected by the UAV for target tracking.	[105]
Hybrid convolutional prediction models	A new model for zero-click action recognition is here studied, which jointly captures the object relations of a static frame and models the temporal motion patterns of adjacent frames.	[106]
	This work develops an innovative framework that integrates ground-based and satellite observations through DL to improve photovoltaic production forecasts.	[107]

### 2.3. Reinforcement Learning-Based Methods 

Over the years, solutions have been devised that do not involve the use of a convolutional neural network. Most of these works exploited deep neural networks in Reinforcement Learning (RL) frameworks applied to the SSA domain. Reinforcement Learning is an area of AI, wherein agents are trained to take decisions or actions by interacting with the environment in order to maximize a return [108].

For example, in [109], authors simulated a controllable ground telescope observing satellites in LEO in an RL environment (Double Deep Q-Learning). Once the number of satellites observed over a period is maximized and the number of successful measurements is increased, they used an extended Kalman filter to significantly reduce the uncertainties of position and velocity measurements for the observed satellites. This work forms the basis of a Reinforcement Learning based framework to be explored in future research in the field of space situational awareness. 

Devices such as Floating Space Manipulators (FFSM) are increasingly used in various space activities, and Active Object Tracking (AOT) is the basis of many space missions. The AOT of FFSM systems presents two major challenges: the modeling and control of FFSM systems and the planning of the tracking motion of spatial manipulators. To address these issues, the paper [110] presented a strategy for active object tracking of FFSM systems employing Deep Reinforcement Learning (DRL), Proximal Policy Optimization (PPO), and a fuzzy neural network in order to address these challenges (FNN). The DRL paradigm does offer a fresh perspective on how to handle challenging space missions, and it works well in conjunction with more conventional approaches. Data acquisition costs, training efficiency, sensitivity to parameters, and environment are just a few of these problems. The algorithm training process was then completed in a simulation environment that is as realistic as possible, where the algorithm was then directly applied to a real physical problem.

Another example of using the DRL can be found in [79]. An internal SSA environment is developed to efficiently train and evaluate the performance of DRL agents. The SSA environment can support arbitrary sensor position, various RSOs, observation windows, and sensor properties (action, change time, stabilization time, dwell time, measurement model, measurement, and process noises). Four DRL agents are trained using population-based training and proximal policy optimization for the SSA sensor tasking problem. Both are fully connected, and convolutional neural network DRL agents are explored in this work. DRL agents are also studied for their resilience to changes in orbital regimes, observation window lengths, observer positions, and sensor change rates. DRL agents have shown solidity to most of these variations and continue to outperform short-sighted policies.

The paper [111] provides the first results to solve the sensor-tasking and sensor-management (SM) problem for Space Situational Awareness (SSA) using the asynchronous advantage Actor Critical Method (A3C), i.e., a Reinforcement Learning (RL) approach. It can learn how to make optimal decisions by interacting with the environment. The A3C method combines the benefits of both of the two main kinds of RL approaches—policy-based and value-based approaches. The A3C approach can also pick up information asynchronously from a collection of agents that engage with the environment. The A3C approach has the advantage of delivering more accurate political gradient estimations with less noise, which can enhance convergence. In this study, the preliminary findings for two simulation cases were presented. The first case involved 100 SOs, and the second case involved 300 SOs. However, results need to be explored further as network optimization has not been explored. 

Finally, we conclude the section with [112], where a fast detection method of space debris with grid-based learning is proposed. The image is divided into 14 × 14 grids, then the fast grid-based neural network (FGBNN) is used to pinpoint the location of the spatial debris in the grids. The proposed method demonstrates excellent performance and high detection speed. FGBNN can process 430 images with an image size of 224 × 224 per second (2.3 ms/image) based on the advanced one-pass framework with few parameters. The experimental results show that this method, characterized by a high detection rate (roughly 98.8% of accuracy) and low computational load, can help detect space debris in applications with a short delay and low power consumption. In Table 3, we summarize the methods, their characteristics, and the bibliographic reference.

**Table 3:** Reinforcement Learning-based methods for SSA space debris detection.

Method Name	Description	Reference Number
Deep Reinforcement Learning	A controllable ground-based telescope observing satellites in LEO is simulated in a reinforcement learning environment.	[109]
	The Proximal Policy Optimization (PPO) method, a fuzzy neural network, and Deep Reinforcement Learning (DRL) were used to demonstrate a proposal for active object tracking of FFSM systems (FNN).	[110]
	For the SSA sensor tasking problem, four DRL agents are trained using population-based training and proximal policy optimization.	[79]
	This paper provides the first results to solve the sensor-tasking and sensor-management (SM) problem for Space Situational Awareness (SSA) using the asynchronous advantage Actor-Critical Method (A3C).	[111]
	A fast detection technique of space debris with grid-based learning is proposed. The image is divided into 14 × 14 grids, then the fast grid-based neural network (FGBNN) is used to pinpoint the location of the spatial debris in the grids.	[112]

## 3. Case Study

**Figure 4:**
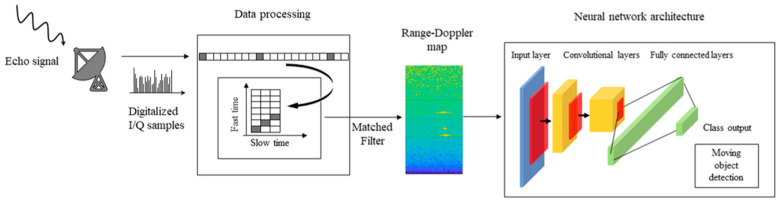
Flowchart of the experimental evaluation presented in this paper.

In this and the following section, we show preliminary results and comparisons by applying deep learning methods for SSA applications in a simulated environment. Although deep learning could be employed for several different tasks, as well as in different stages of a generic processing chain, in our tests, we will focus on a well-defined and paradigmatic use case, i.e., small moving object detection in LEO from radar signals. To do that, we simulate the standard processing chain of a monostatic pulse-Doppler radar that detects the radial velocity of moving targets at specific ranges. The radar output is used to feed a neural network architecture that provides the number of detected targets, as shown in Figure 4.

### 3.1. Radar Processing

At the receiver, the electromagnetic echoes reflected by a target are split into their I and Q (in-phase and quadrature) components by using coherent demodulation. As a propagation form, we assumed a two-rays ground reflection model, while targets have been characterized in terms of distance (i.e., range) from the radar, velocity, and Radar Cross Section (RCS). Once the I and Q components are extracted, digital sampling is performed after a signal windowing that controls the sidelobes level caused by the sampling operation. Afterward, pulse-Doppler signal processing separates reflected signals into channels by means of a set of filters for each ambiguous range. The maximum unambiguous range is related to the inverse of the Pulse Repetition Frequency (PRF) of the radar. More in-depth, the I and Q signals are filtered by the following scheme: the samples are reshaped into a time domain matrix; columns correspond to range samples (fast time), while rows correspond to pulse intervals (slow time). Convolved with the matched filter by means of a Fast Fourier Transform operation, the output matrix provides a power spectral density estimate of the returned signal in function of the range and Doppler frequency. This matrix is also known as a range-Doppler map. In a classical pulse-Doppler radar processing chain, the estimation of target position and velocity is performed by thresholding the range-Doppler map and finding the range and the Doppler bins in which the energy exceeds the given threshold. In our experiments, these maps are the input of the neural network architectures that we want to compare. For further details on radar signal processing, the reader may refer to [113].

### 3.2. Neural Network Frameworks

The range-Doppler maps are then used as inputs for the deep learning frameworks that we use for comparisons. We test convolutional neural networks to work correctly and efficiently with 2D image inputs. The chosen networks were SqueezeNet, VGG-16, AlexNet, and GoogLeNet, adjusting their parameters and levels for this specific use case:

- SqueezeNet is a very light architecture which, nevertheless, achieves outstanding performance in computer vision tasks. The basic idea of the SqueezeNet network is to create a small neural network with few parameters, which can easily adapt to portable devices, thus having a lower computational burden, lower memory demand, and reduced inference time. It is made up of 18 layers. The compression layer consists of 1-by-1 convolutions, which combine all input data channels into a single channel. This procedure reduces the number of inputs for the next level. Data reduction is obtained also by using max-pooling layers, which perform a pooling operation that calculates and retains the maximum value of each patch inside each feature map. In a SqueezeNet, the last learnable layer is the final convolutional layer, unlike in most networks, where the last layer with learnable weights is generally a fully connected layer.
- VGG-16 is a sixteen-layer deep neural network with about 138 million parameters. This implies that it takes a long time to be trained and to make an inference. It also occupies a significant amount of memory (roughly 533 MB). Despite this, it has been used in several image classification problems, and in terms of performance, it provided the best result with a test error of about 7.0%. [114,115]. The main design idea was to increase the depth by using smaller (3 × 3) multiple convolutional filters than those of previous networks. VGG-16 is composed of a stack of 13 convolutional layers followed by three fully connected layers. Each convolutional block consists of multiple convolutional layers, each with different 3 × 3 filter kernels. As the depth increases, the number of filters in the levels grows, from 64 up to 512, in order to extract increasingly detailed feature maps from each block. The convolutional layers are followed by a rectified linear unit activation layer, and each block ends with a maximum pooling layer, with a 2 × 2 sliding filter with step 2. At the end of the network, there are three fully connected layers: the first two layers have 4096 nodes each; the third performs the 1000-way ILSVRC classification and, therefore, contains 1000 output nodes. This final layer comes with a soft-max activation function for classification. Since VGG-16 is computationally demanding and the output layer does not match our case study, we customized it by modifying the last layer. We set the number of output nodes equal to the number of classes that we have defined in our use case (4, as we will describe later in the next section). As a final step, we froze the first ten layers, and we performed new training to learn new network weights for the last layers.
- The AlexNet architecture consists of five layers with a combination of max pooling, followed by three fully connected layers [116]. It uses rectified linear units instead of a hyperbolic tangent function. The advantage is twofold: it is faster to compute, especially during network training, and it mitigates the problem of vanishing gradients [117]. An interesting property of this network is that it allows parallel-GPU training by placing half of the model neurons on one GPU and the other half on the second one. This allows us to train larger models or to reduce training time. Moreover, dropout layers are used to avoid overfitting. This technique works by randomly firing a set of neurons within the first two fully connected layers during the whole training. The price to pay is that it increases the training time required for model convergence. AlexNet demonstrated significantly higher performance by achieving high accuracy on very challenging datasets, and it can be credited with bringing deep learning to other fields, such as natural language processing and medical image analysis [118].
- GoogLeNet, developed by Google, was responsible for creating a new state of the art for classification and detection tasks in the ILSVRC. It also has been used for other computer vision activities, such as face detection and recognition, or in adversarial training. GoogLeNet’s architecture is 22 levels deep, with 27 pool levels included. There are nine starter modules stacked linearly. The ends of the startup modules are connected to the global average pooling level. Since neural networks are time-consuming and expensive to train, the number of input channels is limited. The first level of convolutions uses a filter size of 7 × 7, which is relatively large compared to other kernels within the network. The main purpose of this layer is to immediately reduce the input image without losing spatial information. To address the overfitting problem, the GoogLeNet architecture was created with the idea of having multi-dimensional filters that can operate on the same level. With this idea, the network becomes wider rather than deeper. For ILSVRC 2014, GoogLeNet ranked first with an error rate of 6.67% [119].

## 4. Results and Discussions

**Figure 5:**
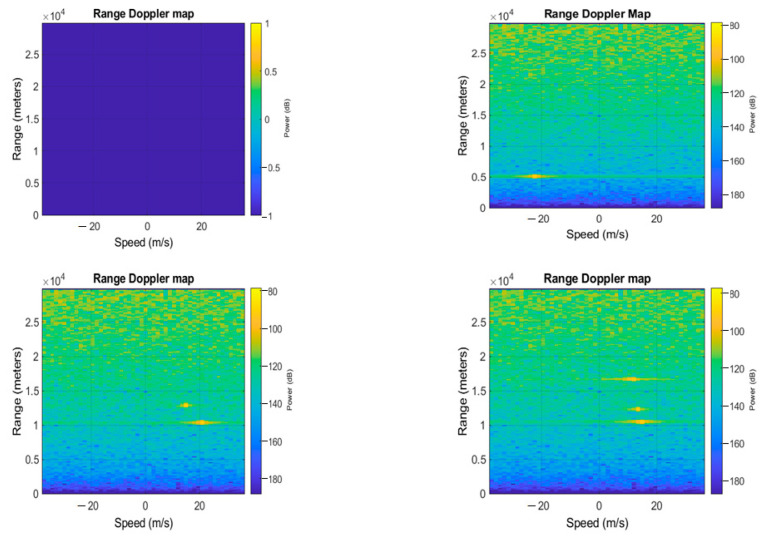
Example of range-Doppler maps generated with (respectively, from the upper left image to the lower right image) 0, 1, 2, 3 targets.

**Figure 6:**
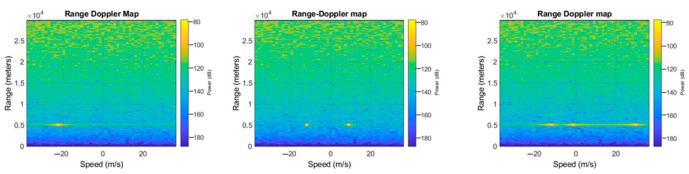
Example of range-Doppler maps generated with fixed positions at different speeds of (respectively, from the left to right) 1, 2, 3 targets.

**Figure 7:**
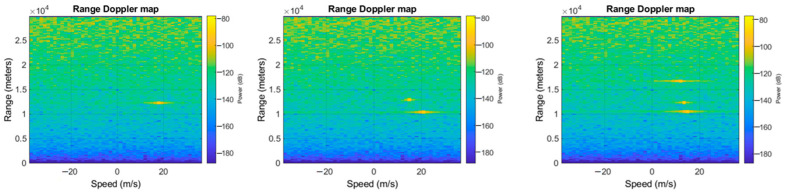
Example of range-Doppler maps generated with speeds of 225 m/s and different positions of (respectively, from the left to right) 1, 2, 3 targets.

In order to carry out our experiments, we have built a dataset that has been structured as follows: range-Doppler maps have been generated as the position and speed varied, and by assigning to each spectrum one of four possible labels, where each represented the number of targets detected (zero if there are no objects, one if there is only one target and so on). Once the images were generated, they were given as input to neural networks for classification. The networks, therefore, classify whether the image, when the position of the object and the speed at which it moves varies, belongs to the first, second, third, or fourth case mentioned. Figure 5, Figure 6 and Figure 7 show the range-Doppler maps in presence of no object, and one, two, and three objects moving. The positions of each target change from a range from 1 to 3000 m, and the maximum detectable speed is 225 m/s.

The entire dataset consists of 800 images, and it was exploited as follows: 640 images (80% of the dataset) were used for the training phase, while 160 images (20% of the dataset) were used for testing the networks. In addition, we used the stochastic gradient descent with momentum (SGDM) optimizer, and the learning rate is 0.0003 with 50 epochs and 50 batch sizes. Hyperparameters are chosen by looking at the literature experiences and best practices. In this sense, hyperparameter optimization is out of the scope of this study. Nevertheless, it should be explored in the future and extended works. 

**Figure 8:**
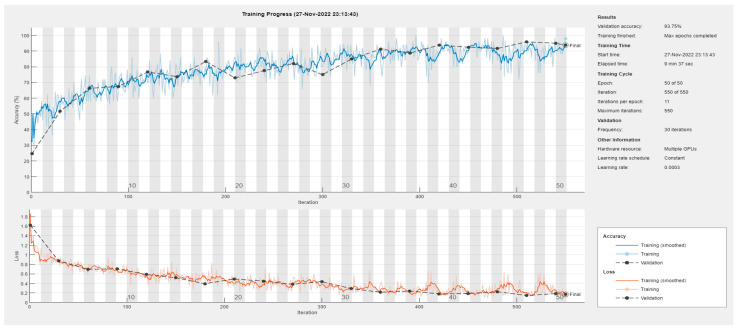
Learning curves of the SqueezeNet DL method.

As an example, Figure 8 illustrates the training curves of the SqueezeNet network (the learning curves of the other approaches are not reported here for the sake of space).

To understand the performance of the considered deep learning classifiers, we have exploited the following metrics:

(1) $$\mathrm{Accuracy}=\frac{\mathrm{TP}+\mathrm{TN}}{\mathrm{TP}+\mathrm{TN}+\mathrm{FP}+\mathrm{FN}}$$

(2) $$\mathrm{Precision}=\frac{\mathrm{TP}}{\mathrm{TP}+\mathrm{FP}}$$

(3) $$\mathrm{Recall}=\frac{\mathrm{TP}}{\mathrm{TP}+\mathrm{FN}}$$

(4) $$F-\mathrm{Measure}=\frac{2\;\mathrm{Precision}\;\mathrm{Recall}}{\mathrm{Precision}+\mathrm{Recall}}$$

True positives (TP) are the number of samples correctly classified as belonging to its true class, while true negatives (TN) are the number of samples correctly classified as not belonging to a given class. False positives (FP) are the number of samples misclassified as coming from a given class, but they belong to another class. On the contrary, false negatives (FN) are the number of samples classified as belonging to a class that does not correspond to the true class. Accuracy, as depicted in (1), is a function of these quantities and it provides an intuitive way to determine which model is best at classifying input data. Furthermore, the better a model can generalize the ‘unseen’ data, the better predictions and insights it can produce. Precision, (2), quantifies the number of positive class predictions that belong to the positive class, while recall, as noted in (3), is the number of positive class predictions obtained from all positive examples in the dataset. The advantage of using precision and recall is that they can fairly describe classification performance also in presence of unbalanced datasets. Finally, the F-Measure in (4) provides a single score that balances both the concerns of precision and recall in one number.

**Figure 9:**
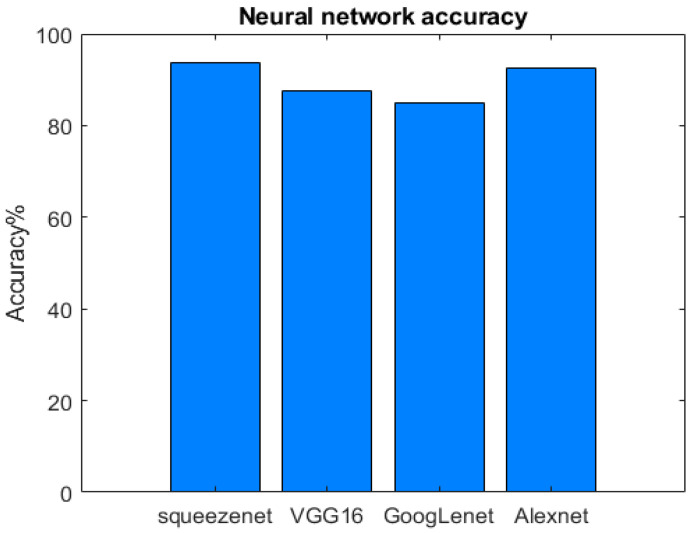
Overall accuracy of DL Networks in object detection for SSA applications.

In Figure 9, we can see the results in terms of the overall accuracy of the SqueezeNet, VGG-16, GoogLeNet, and AlexNet networks. The obtained results are very good, and all oscillate around 85–95%. Finally, in Table 4, we show the results in terms of precision, recall, and F-measure for each considered network.

The SqueezeNet was found to be the best-performing network in terms of overall accuracy. In addition to being a “squashed” network, therefore with much fewer parameters, it was the fastest to train. The AlexNet network follows in terms of performance. Using CNN with fewer layers has the advantage of lower hardware needs and shorter training times than VGG-16 and GoogLeNet. Indeed, shorter training times allow for testing more hyperparameters. This makes the entire training process easier. One of the key design choices of the VGG-16 network was to use smaller (3 × 3) convolutional filters than those of previous networks. This allows an accurate recognition of the images (with an overall accuracy of 87.50%, as we can see in Figure 9), at the cost of dramatically increasing the depth of the network. the VGG-16 model is an extremely heavy net (thanks also to its depth and the number of fully connected layers), characterized by the slowest training times. GoogLeNet network, although it has reached a good accuracy (85%), has turned out to be the least performing, but still faster than the VGG-16 (which exceeds by only 2% accuracy).

## 5. Conclusions and Future Directions

Space infrastructures are subject to collisions with debris, abandoned space objects, and other active satellites every day. This is a big problem as modern society relies heavily on space infrastructure for day-to-day operations, such as communication, guidance, navigation, weather forecasting, and spatial images. Therefore, being aware of the spatial situation and developing further new algorithms for the defense of space infrastructures is very important. This review paper focused on such dramatic issues, reviewing the recent papers published in the field of machine and deep learning for object detection in satellite-based Internet of Things (SIoT) networks. In particular, the first part of our paper discussed the importance of SIoT, explaining what it is, why it is important, and its main uses in everyday life. Then, we have illustrated the problem related to space debris and the reasons why the protection of satellites’ constellations is fundamental and necessary. The main methods of object detection were shown first without the use of deep learning frameworks and then with the use of CNN, which proved to be the best network to tackle this type of problem. In addition, innovative approaches were shown with other typologies of networks, discussing the use of Deep Reinforcement Learning. Finally, we conducted a comparison between several DL techniques for object detection in SSA scenarios via simulation results. Four different DL frameworks, namely Squeeze Net, Google Net, VGG-16, and Alex Net, were used for object detection for SSA applications. In particular, we simulated the use of a monostatic pulse radar that detects the radial velocity of moving targets at specific intervals. The results obtained through simulations demonstrate the efficiency of such methods for object detection in SSA scenarios, thus improving the SIoT network resilience to collisions and damages with space debris.

Radar and optical systems are both used in space surveillance. However, their performance and scope are very different. On the one hand, optical telescopes can observe objects at a great distance if the conditions are suitable and the angular velocities are low, but their efficiency highly depends on good weather conditions and adequate lighting during the night hours. On the other hand, radars are capable of tracking objects with higher angular velocities; they are available 24/7 and without restrictions of weather conditions. However, their sensitivity depends on the distance; radars are not very effective at tracking objects in higher positions compared to MEO (Medium Earth Orbit). Deep learning methods have been fully applied in radar detection. Using DL can not only process large amounts of data but also minimize or eliminate redundant and unimportant data. In addition, satellite imagery is ubiquitous in space, and networks such as CNN are especially good for working with imagery. Thus, characteristics that may not be identifiable to a human can be efficiently extracted by DL approaches. Furthermore, these approaches surpass traditional techniques in terms of accuracy. Not only DL models, such as radars, are robust to any meteorological change, but they also can work on any data, be it structured, unstructured, or semi-structured. The fusion of measurements from optical telescopes and the radar united with DL methodologies should provide highly acceptable orbit determination results because it is possible to combine the respective advantages and compensate for the respective disadvantages. 

The authors believe that in the next future, unfortunately, the conflict between the required performance, the number of sensors, and the growing need for SSA data acquisition will continue to exist. The optimization of the system’s understanding capacity promises to reduce this conflict, as well as the multisource data fusion. Then, several important shortfalls must be counteracted in the next future, such as the need for large training datasets and very high training times. Hence, the designing of new classifiers is now moving towards the choice of exploiting heterogeneous data in small sample scenarios, thus reducing the training times and the requirements for the requested computational complexity. Finally, it’s the authors’ opinion that another problem that will be faced by researchers in the future would be the need to work with short monitoring intervals—being able to react quickly to debris decomposition for satellite maneuvers will be the next research topic area, thus improving the capacity of non-stop orbital prediction, tracking, and monitoring in hazardous situations. Hence, research will be conducted in order to strengthen the confidence of the system by improving early warning collision systems, as well as studying more accurate maneuvering avoidance policies.

## Figures and Tables

**Table 4:** Precision, Recall, and F-measure of DL Networks for SSA space debris detection.

	Squeeze Net	VGG-16	Google Net	Alex Net
Precision	0.937	0.875	0.852	0.925
Recall	0.94	0.885	0.855	0.932
F-measure	0.937	0.872	0.842	0.928

## Data Availability

No new data were created or analyzed in this study, Data sharing is not applicable to this article.

## Abbreviations

**Acronym**	**Explanation**
A3C	Actor-Critical Method
AI	Artificial Intelligence
AIOT	Artificial Intelligence of Things
AMIGO	Adaptive Markov Inference Game Optimization
ANN	Artificial Neural Network
AOT	Active Object Tracking
ASPP	A parallel Spatial Pyramid Pooling
CCTV	Close-Circuit Television
CNN	Convolutional Neural Network
DL	Deep Learning
DNN	Deep Neural Network
DRL	Deep Reinforcement Learning
FC	Fully Connected
FCC	Federal Communications Commission
FCN	Fully Convolutional Network
FFSM	Floating Space Manipulators
FFT	Fast Fourier Transform
FGBNN	Fast Grid-Based Neural Network
FNN	Fuzzy Neural Network
FOV	Field of View
ILSVRC	ImageNet Large Scale Visual Recognition Challenge
IOT	Internet of Things
ISS	International Space Station
K-NN	k-Nearest Neighbor
K-NN-DTW	k-Nearest Neighbor combined with Dynamic Time Warping
LEO	Low Earth Orbit
LSTM	Long-Short Term Memory
MCNNT	Modified Convolutional Neural Networks Technique
MEO	Medium Earth Orbit
MF-TBD	Multi-Frame Track-Before-Detect
ML	Machine Learning
PPO	Proximal Policy Optimization
PRF	Pulse Repetition Frequency
RCS	Radar Cross Section
RELU	Rectified Linear Unit
RL	Reinforcement Learning
RNN	Recurrent Neural Network
ROI	Region of Interest
SDA	Space Domain Awareness
SGDM	Stochastic Gradient Descent with Momentum
SIOT	Satellite Internet of Things
SM	Sensor Management
SNR	Signal to Noise Ratio
SSA	Space Situational Awareness
STM	Space Traffic Management
SVM	Support Vector Machine
TDNN	Time-Delay Neural Network
UAV	Unmanned Aerial Vehicles
